# Tregs and Mixed Chimerism as Approaches for Tolerance Induction in Islet Transplantation

**DOI:** 10.3389/fimmu.2020.612737

**Published:** 2021-01-29

**Authors:** Shiva Pathak, Everett H. Meyer

**Affiliations:** ^1^Division of Blood and Marrow Transplantation, Stanford University School of Medicine, Stanford, CA, United States; ^2^Stanford Diabetes Research Center, Stanford University School of Medicine, Stanford, CA, United States

**Keywords:** Tregs, islet transplantation, hematopoietic stem cells, mixed chimerism, transplant tolerance

## Abstract

Pancreatic islet transplantation is a promising method for the treatment of type 1 and type 3 diabetes whereby replacement of islets may be curative. However, long-term treatment with immunosuppressive drugs (ISDs) remains essential for islet graft survival. Current ISD regimens carry significant side-effects for transplant recipients, and are also toxic to the transplanted islets. Pre-clinical efforts to induce immune tolerance to islet allografts identify ways in which the recipient immune system may be reeducated to induce a sustained transplant tolerance and even overcome autoimmune islet destruction. The goal of these efforts is to induce tolerance to transplanted islets with minimal to no long-term immunosuppression. Two most promising cell-based therapeutic strategies for inducing immune tolerance include T regulatory cells (T_regs_) and donor and recipient hematopoietic mixed chimerism. Here, we review preclinical studies which utilize T_regs_ for tolerance induction in islet transplantation. We also review myeloablative and non-myeloablative hematopoietic stem cell transplantation (HSCT) strategies in preclinical and clinical studies to induce sustained mixed chimerism and allograft tolerance, in particular in islet transplantation. Since T_regs_ play a critical role in the establishment of mixed chimerism, it follows that the combination of T_reg_ and HSCT may be synergistic. Since the success of the Edmonton protocol, the feasibility of clinical islet transplantation has been established and nascent clinical trials testing immune tolerance strategies using T_regs_ and/or hematopoietic mixed chimerism are underway or being formulated.

## Introduction

Type 1 diabetes (T1D) arises from an autoimmune attack of the insulin-producing, islet beta cells of the pancreas. Patients with T1D exhibit abnormalities in immune regulation that contribute to its etiology. Organ/tissue transplantation is complicated by adaptive CD4^+^ and CD8^+^ T cell responses that can contribute to allograft rejection (1–4). Owing to the combined specters of auto- and allo-immune responses, islet transplantation is one of the most challenging settings to prevent immune rejection.

Pharmacologic immunosuppressive drugs (ISDs) in islet transplantation traditionally target effector T cell proliferation and function to prevent graft rejection (5). However, most of these ISDs require life-long administration and have increased risk of multiple adverse reactions, including susceptibility to infection and incidence of secondary cancers (6, 7). In addition, survival of the transplanted islets is shortened due to direct toxic effects of the ISDs on islet β cells (8). One of the major goals in islet transplantation is the induction of immunosuppressive drug-free tolerance to the islet graft (9–11).

By virtue of their role in controlling alloreactive T cell responses to organ and tissue grafts, regulatory T cells (T_regs_) are considered as promising alternatives to pharmacologic agents to promote engraftment and survival of the transplanted organs/tissues (12–14). Peripheral tolerance established by T_regs_ is crucial to prevent immune-mediated rejection of the transplanted graft (15, 16). Several preclinical studies have demonstrated induction of immune tolerance in different transplantation models such as heart, kidney, skin, liver, and islets (17–20). Multiple clinical trials are in progress evaluating the efficacy of recipient T_regs_ in organ transplantation tolerance (clinicaltrials.gov). One promising strategy in preclinical studies is the adoptive transfer of *in vitro* culture expanded T_regs_ to prevent the rejection of donor islet grafts (21, 22) and at least one clinical trial testing this approach is underway (NCT03444064). This phase I clinical trial aims to assess the safety and feasibility of autologous polyclonal T_regs_ in islet transplant patients. However, pre-clinical studies and clinical studies with recipient-derived T_regs_ in solid organ transplantation have shown that peripheral T cell tolerance is not necessarily durable and methods to enhance T_reg_ function is an active area of research.

**Figure 1:**
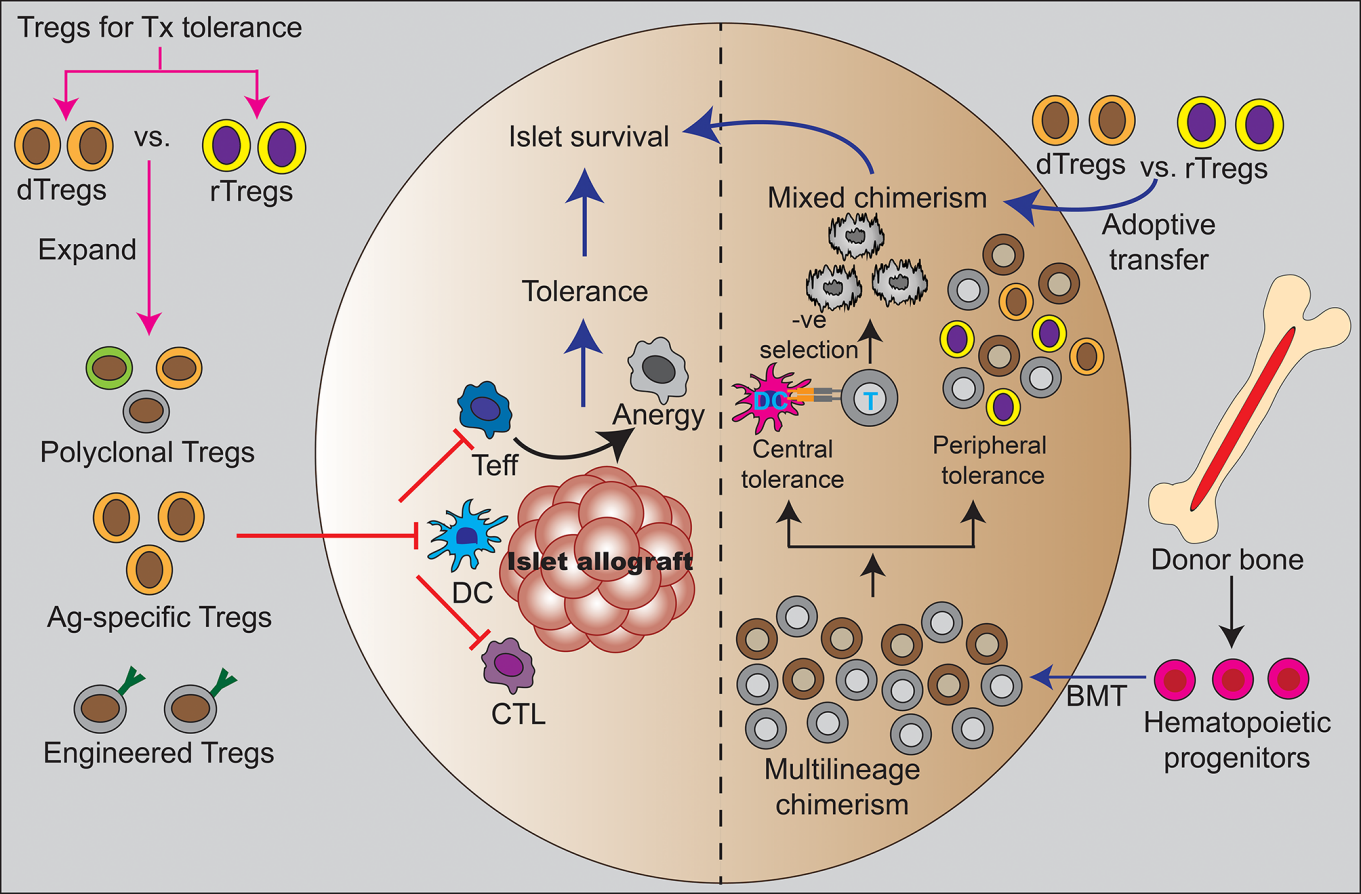
T_reg_ and hematopoietic mixed chimerism as clinical strategies for tolerance induction. The left half of the figure shows direct effect of T_regs_ in inducing peripheral tolerance by regulating different immune cells such as dendritic cells and T cells to suppress alloreactivity. The adoptive transfer of different types of T_regs_ that been used in preclinical studies to support mechanisms of peripheral islet tolerance including polyclonal T_regs_, antigen-specific T_regs_, and engineered T_regs_. These studies suggest T_regs_ might be used to reduce or eliminate systemic immunosuppression. The right half shows establishment of mixed hematopoietic chimerism through combined donor islet and hematopoietic stem cell transplantation. This is a state of coexistence of donor and recipient hematopoietic cell precursors with evidence to indicate that both mechanisms of central deletion of alloreactive responses and peripheral tolerance pathways regulate allograft tolerance. The administration of exogenous T_regs_ have been used to promote mixed hematopoietic chimerism and tolerance in preclinical studies. T_reg_ are necessary for sustained chimerism and tolerance in these models and human clinical studies have shown T_reg_ exert allo-antigen specific regulation in the setting of mixed chimerism. Ag, antigen; BMT, bone marrow transplantation; CTL, cytotoxic T lymphocytes; DC, dendritic cell; dTregs, donor-derived regulatory T cells; rTregs, recipient-derived regulatory T cells; T, T cell; Teff, effector T cell.

Another cell-based strategy for inducing islet allograft tolerance originates from studies which showed that the establishment of hematopoietic mixed chimerism between the donor and recipient results in donor allograft tolerance (**Figure 1**) (23, 24). Subsequent preclinical islet transplantation models which rely on mixed chimerism for tolerance induction have developed clinically translatable approaches (25–28). Encouragingly, clinical trials of combined kidney and hematopoietic cell transplantation (HCT) from living donors have demonstrated that immune tolerance to solid organs is possible by establishing hematopoietic mixed chimerism. Over 80% of HLA-matched patients enrolled in these trials are completely off ISDs (29, 30).

In the case of islet transplantation for T1D, experiments in preclinical murine models first reported almost 20 years ago have reproducibly shown that the establishment of hematopoietic mixed chimerism not only provides durable allograft tolerance but also prevents autoimmune islet destruction (31). A major problem in the translation of combined islet and HCT has been the traditionally toxic conditioning required for HCT, but the bone marrow transplantation field is rapidly evolving and significantly less toxic approaches have been developed or are in early phase clinical trials (32–34). Thus, combined islet and HCT is a promising area of translational investigation.

Since T_regs_ play a critical role in the establishment of tolerance in the setting of hematopoietic mixed chimerism (**Figure 1**), it is important to better understand T_regs_ in this setting. It is also possible that a combined immune therapy of T_regs_ and HCT may be synergistic (35).

## Tregs in Autoimmune Diabetes

T_regs_ are a small subsets of CD4^+^ T cells, characterized by the surface expression of CD4 and CD25, and the expression of the transcription factor forkhead box protein 3 (Foxp3) which is critical for their function (36). T_regs_ are well-known for their suppressive function and are responsible for safeguarding against various autoimmune diseases, including T1D (37, 38). This review focuses on major exogenously-administered T_regs_ that have been used in bone marrow and islet transplantation settings with a special emphasis on the classical CD4^+^CD25^+^Foxp3^+^ T_regs_.

Non-obese diabetic (NOD) mice spontaneously develop autoimmune diabetes and share many features of human T1D (39, 40). Preclinical studies in NOD mice have shown that T_reg_ can prevent autoimmune diabetes (41–43). The NOD mice which have defective CD28/B7 costimulation pathway and are prone to exacerbated T1D pathology showed delayed diabetes progression when injected with CD25^+^ T_regs_ (44). Moreover, adoptive transfer of islet-specific T_regs_ reversed T_reg_ defect in CD28 deficient NOD mice and successfully prevented the disease progression (45). These series of findings suggest ex vivo-expanded T_regs_ as a way to satisfy T_reg_ deficiency in the treatment of T1D.

T1D is characterized by presence of defective T_regs_ function and activation, particularly in the IL-2 pathway which can also affect T_reg_ function (44–47). The role of IL-2 signaling in T_reg_ development, metabolism, and function has been discussed in a recent review (48). Defective IL-2 signaling is associated with impaired T_reg_ metabolism and diminished suppressive function (49). Ex vivo expansion of T_regs_ derived from T1D patients may be a principle way to correct for any inborn deficiency and these T_regs_ have been tested for their safety in phase 1 clinical trials with no evidence of therapy-related adverse events reported (50). Another clinical study has shown similar safety in pediatric T1D patients, and suggests disease modulation with observed reduced daily insulin requirement in treated patients (51, 52).

## Tregs for Promoting Islet Engraftment

T_reg_ therapy can be applied in two settings in islet transplantation: promoting islet survival in initial engraftment and inducing peripheral tolerance to eliminate immunosuppression.

The most common implantation site for clinical islet transplantation is within the liver *via* hepatic portal vein infusion (53). It is estimated that >50% of the initial islet mass that is infused is lost within the first few days due to local inflammatory changes and coagulation at the islet implantation site; the phenomena is termed as instant blood-mediated inflammatory reaction (IBMIR) (54, 55). The addition of T_regs_ at the time of islet infusion has been explored as a method for reducing initial islet graft loss and improving islet engraftment (56–58).

In addition to potentially changing the inflammation at the islet implantation site, experiments in which T_regs_ are either co-cultured, co-aggregated, or co-infused with islets have shown that T_regs_ appear to affect the islets themselves (59–62). In a preclinical study, co-culture of T_regs_ with the pancreatic islets altered production of inflammatory chemokines such as CCL2, CCL5, CXCL9, and CXCL10, produced by the islets themselves, benefitting islet graft survival after implantation under the kidney subcapsule (60). TGF-β secreted from the T_regs_ have been shown to improve islet viability and function in islet-T_reg_ coculture experiment (63). T_regs_ might therefore improve islet viability and potentially reduce their immunogenicity.

A number of studies have provided preclinical evidence that T_regs_ incorporated into the islet graft itself or co-administered with the islet allograft can inhibit adaptive immune responses (59, 61, 64). In one example, Takemoto et al. constructed co-aggregates of BALB/c islets and C57BL/6 T_regs_ and transplanted into the liver of C57BL/6 mice where a long-term survival of the allogenic islets was observed for over 100 days without any immunosuppression (65).

## Tregs for Modulating Adaptive Responses in Islet Transplantation

Adoptive transfer of recipient-derived T_regs_ in preclinical models has shown to be effective in preventing islet allograft rejection through the establishment of transplant tolerance (**Figure 1**). Polyclonal T_regs_ have been used either to protect islets from direct contact-mediated immune attack or to modulate systemic immune response (59–61, 65, 66). Zhang et al. adaptably transferred donor antigen-specific T_regs_ in mice and found a profound synergistic effect with rapamycin in the islet allograft transplant setting (67). In another study by Lee et al, adoptive transfer of donor-reactive T_regs_ in T cell depleted mice resulted in indefinite survival of islet allografts. Moreover, *in vitro* expanded T_regs_ have been shown to delay porcine islet xenograft rejection in humanized mice by inhibiting graft-infiltrating effector T cells (64). In addition, multiple studies have demonstrated that local co-injection of islets and T_regs_ promotes islet engraftment (59, 60, 65).

## Choice of Tregs in Islet Transplantation

An essential question remains unanswered in studies that examine the use of T_reg_ therapy: which is better, donor or recipient T_reg_? This has perhaps been shaped by the perception that the only available source of clinical-grade T_regs_ is from the recipient, but T_regs_ can potentially be obtained from cadaveric spleen and bone marrow for clinical use.

It would be reasonable to hypothesize that both donor and recipient T_regs_ may reduce inflammation during islet transplantation. Perhaps donor T_regs_ would be more effective because of alloreactive responses to recipient MHC Class II expressed by local APCs or other cells. Likewise, recipient T_regs_ co-cultured with islets themselves might be more effective in changing islet profiles, as recipient T_regs_ may be more able to exert effector function through alloreactive TCR responses.

In case of T_reg_ modulatory effects on adaptive immune responses, recipient T_regs_ might be favored as their initial alloreactive responses to islet tissue could locally shape the recipient adaptive immune response to allow alloreactive T_regs_ to persist and expand. Alternatively, donor T_regs_ might be more able to modulate adaptive responses by early and critical interactions with infiltrating recipient immune cells. In T1D patients, it is possible that recipient T_regs_ may also have deficiencies that could be avoided with the use of donor T_reg_ therapy, however other methods such as ex vivo expansion or genetic modification of T1D T_regs_ are being explored (68–70). Alternatively, both donor or third-party T_regs_ could be utilized.

## Improving Treg Function

Outside of HCT, clinical trials with T_regs_ have generally shown an excellent safety profile but generally unclear efficacy. This may be because many studies do not use lymphodepletion, which may help with T_reg_ engraftment (71, 72). Furthermore, the persistence of T_regs_ may be affected by the concomitant use of immunosuppressive drugs (73), with some evidence pointing to low dose IL-2 and rapamycin as a more effective strategy than other immunosuppressive regimins (74). Likewise, the use of low dose IL-2 and protein engineered IL-2 derivatives is being explored (75). Other promising methods of inducing T_regs_ *in vivo* such as the administration of tolerogenic CD11c^+^ DCs or pharmacologic stimulation of T_reg_ are well described elsewhere (76–78).

Gene modification techniques have been proposed as alternative strategies to produce more active and efficacious T_regs_ in a large scale, involving two approaches: engineering T_regs_ with T cell receptor (TCR) (79–81) or chimeric antigen receptor (CAR) (82–84). Islet antigen-specific T_regs_, generated using lentiviral-mediated TCR gene transfer, were capable of inhibiting effector T cells through antigen-specific suppression (81). This demonstrates the potential applicability of islet antigen-specific T_regs_ in the prevention of diabetes progression as well as in islet transplant settings. Islet antigen-specific T_regs_ generated using lentiviral transduction showed strong suppressive activity in an antigen-specific manner, providing a proof-of-concept for the potential use of TCR gene transfer technology-enhanced T_reg_ activity in islet transplantation (81). Thus, gene transfer technology is likely to be adapted to enhance the therapeutic efficacy of T_regs_ while avoiding the pan-immunosuppression effect of polyclonal T_regs_.

CAR T_regs_ are genetically engineered cells which express single chain variable fragment that recognizes specific antigen on target cells in an TCR-independent fashion. Recently, CAR T_regs_ have received growing attention in different transplantation models (84). Insulin-specific CAR T_regs_ generated using retroviral transduction were shown by Tenspolde et al. to be functionally stable and suppressive *in vivo* (85). The adoptive transfer of ex vivo expanded recipient T_regs_ transiently expressing CAR to target the MHC-I of donor islets in murine models showed improved initial allograft engraftment and survival, with donor-specific tolerance mechanisms observed (86). These studies suggest CAR T_regs_ could exert site-specific and localized immunosuppression.

## Combined Islet and Bone Marrow Transplantation

The use of bone marrow to induce donor-specific tolerance has been tested in different solid organ transplantation models in preclinical and clinical studies in living donor transplantation (87–89). In a seminal study nearly 20 years ago, Sykes showed in murine models that immunological tolerance to allogeneic islets could be achieved in NOD mice with established disease through the bone marrow mixed chimerism across MHC barriers generated using a non-lethal dose of irradiation and a combination of anti-CD4, anti-CD8, anti-Thy1.2, and anti-CD40L mAbs (31). Since then, a number of studies have explored different conditioning regimens including those with different radiation doses or chemotherapy (fludarabine, cyclophosphamide, or busulfan) without irradation (26, 90, 91). A common thread to these studies, lymphodepletion was generally required for alloreactive graft tolerance and sustained chimerism (92).

One remarkable finding of a number of studies that explored NOD recipients is that the mixed chimerism induced from donors was sufficient to overcome autoimmune islet attack. Zeng et al. explored how the degree of MHC mismatch might affect autoimmunity. They showed that increased MHC mismatch from NOD recipients more effectively protects against autoimmune islet rejection (93). It is likely that human cadaveric donors of human islets will be HLA-mismatched, a major question in the clinical translation is which HLA alleles might be overlapping or not.

Oura et al. evaluated islet allograft survival in non-human primates using MHC-mismatched cynomolgus monkeys and found that islet allograft rejection is prevented as long as mixed hematopoietic chimerism is obtained. This is different from tolerance to kidneys transplanted into the same monkeys that were obtained even with a transient mixed chimerism (25, 94). This suggests that islet allografts may be more immunogenic or complicated than solid organ allografts in terms of tolerance induction in humans.

## Complications of Hematopoietic Cell Transplantation Limiting the Application of Mixed Chimerism

One of the major issues with bone marrow transplantation is the intensity of conditioning which has evolved over the past decades with some approaches such as the use of total lymphoid irradiation and antithymocyte globulin (TLI/ATG) have a good safety profile in combined organ and HCT (95). Current developments in safer conditioning in sickle cell are also being explored (96, 97). One of the most promising approach for newer and far less toxic HCT is the use of monoclonal antibodies against hematopoietic stem cell niche constituents instead of radiation or chemotherapy that is now being explored in patients with immune deficiency (98–100). Another complication is graft versus host disease (GVHD) (101) which in part is in large part mediated by donor T cells (102, 103). Early trial results of an ongoing phase 2 clinical trial of T_reg_ therapy given at the time of HCT reinforce their capacity to prevent GVHD (104, 105). Aside from GVHD, dysregulated immunity can be a complication of GVHD which can include viral reactivation of cytomegalovirus (CMV) or Epstein bar virus (EBV) as well as susceptibility to pathogens or opportunistic infections (106, 107). Studies in the HCT setting have not shown an increased risk of viral reactivation with T_reg_ therapy. T_reg_ may help to regulate viral latency (108). In combined kidney and HCT studies in the HLA-matched and haploidentical setting, CMV reactivation might occur more frequently than in kidney transplant alone and appears controlled with antiviral medications (95), however the risks of immune dysregulation in the fully HLA-matched deceased donor setting remains unknown.

## Critical Role of Tregs in Mixed Chimerism and Graft Tolerance

Following bone marrow transplantation, T_regs_ have been used to to prevent GVHD and to prolong allograft survival through the induction of mixed chimerism in combined marrow and organ transplantation (109). In this regard, studies report development of transplant tolerance by T_regs_ in the setting of mixed chimerism (35, 110), the dependency of tolerance on the presence of recipient T_regs_ (111), as well as the need for donor T_regs_ to prevent GVHD (112). In our murine studies, recipient T_regs_ have been shown to promote hematopoietic engraftment after HCT (86). Multiple other studies have shown that the addition of T_regs_ to conditioning increases donor hematopoietic engraftment (105, 113–115). It is interesting to observe a long-term graft tolerance even with the incorporation of T_regs_ which disappear shortly after infusion (73, 116). These findings suggest that the long-term graft survival might be due to the ability of the transferred T_regs_ to induce infectious tolerance. Recent studies show that both the donor and recipient T_regs_ contribute to suppress the alloreactive responses after HCT (105, 117, 118). The integration of T_reg_ therapy into combined organ or islet transplantation is therefore a potentially non-toxic method for improving tolerance induction and establishing mixed hematopoietic chimerism. We are currently testing this in an ongoing trial in combined kidney and HCT from living donors (NCT03943238).

Finally, in the context of islet transplantation with concomitant HCT to induced mixed chimerism, donor T_regs_ are likely more effective in preventing graft-versus-host disease (GVHD) based on preclinical models in which donor T_regs_ were found to prevent GVHD when given at the time of HCT (112).

## Clinical Trials With Tregs

Clinical islet transplantation for T1D patients with severe hypoglycemia unawareness is an approved therapy in the majority of advanced nations (119). This population has severe morbidity and mortality; therefore, clinical trials are needed. Clinical trials integrating T_reg_ therapy and/or hematopoietic mixed chimerism into islet transplantation have been limited. An ongoing clinical trial (NCT03444064) is testing the integration of autologous polyclonal T_regs_ in T1D patients who are receiving the conventional Edmonton islet transplantation protocol. Another clinical trial (NCT03162237) of islet xenotransplantation is currently underway and involves transplantation of 10,000 islet equivalent (IEQ) of porcine islets and infusion of 2 million/kg autologous T_regs_ in the recipients receiving induction immunotherapy with belatacept and maintenance immunotherapy with tacrolimus and mycophenolate mofetil. In the only report of combined islet and hematopoietic transplantation, a small six patient phase 1 trial integrating an infusion of cadaveric hematopoietic stem cells intravenously post-transplant did not successfully lead to donor chimerism or graft tolerance, but showed that the infusion of bone marrow cells from a cadaveric source is safe and potentially feasible (120).

## Conclusion and Future Perspectives

Since the success of the Edmonton protocol in showing the benefit of islet transplantation to patients with hypoglycemia unawareness, the major challenge of achieving and maintaining tolerance remains. The integration of cell therapy approaches such as T_reg_ therapy, mixed hematopoietic chimerism, or a combination of both remain promising.

## Author Contributions

All authors contributed to the article and approved the submitted version.

## Funding

This study was supported by the Leona M. and Harry B. Helmsley Charitable Trust, Juvenile Diabetes Research Foundation United States of America, and the National Institute of Diabetes and Digestive and Kidney Diseases (grant no. P301P30DK11607401).

## Conflict of Interest

The authors declare that the research was conducted in the absence of any commercial or financial relationships that could be construed as a potential conflict of interest.
